# Helicobacter pylori infection in family members of patients with gastroduodenal symptoms. A cross-sectional analytical study

**DOI:** 10.1590/1516-3180.2017.0071311217

**Published:** 2018-06-04

**Authors:** Ayse Palanduz, Levent Erdem, Birsen Durmaz Cetin, Nuran Gülgün Ozcan

**Affiliations:** I MD. Associate Professor, Department of Family Medicine, Istanbul University, Istanbul Faculty of Medicine, Istanbul, Turkey.; II MD. Professor, Department of Gastroenterohepatology, Istanbul Bilim University Faculty of Medicine, Istanbul, Turkey.; III MD. Professor, Department of Infectious Diseases and Clinical Microbiology, Koc University Faculty of Medicine, Istanbul, Turkey.; IV MSc. Biologist, Ministry of Health, Second Public Health Laboratory, Istanbul, Turkey.

**Keywords:** Helicobacter pylori, Disease transmission, infectious, Family, Parents, Siblings

## Abstract

**BACKGROUND:**

Primary *Helicobacter pylori (H. pylori)* infection is acquired predominantly in childhood in the family setting. We aimed to investigate the presence of intrafamilial concurrent *H. pylori* infection.

**DESIGN AND SETTING:**

Cross-sectional analytical study with a control group, conducted in a tertiary care hospital.

**METHODS:**

Fifty adult patients with gastroduodenal symptoms who underwent gastroscopy (index parents), their spouses and their children were enrolled in the study. Blood samples were collected from all of the study subjects to test for immunoglobulin G (IgG) antibody response. *H. pylori* antigen was investigated in the stool specimens of children only.

**RESULTS:**

The participants were divided into two groups: Group 1 consisted of the 40 patients in whom *H. pylori* infection was demonstrated via endoscopy, their spouses and their children. Group 2 included the remaining 10 patients who underwent endoscopy revealing negative results for *H. pylori*, their spouses and their children. IgG antibodies were present in all of the index parents, 95% of their spouses and 93% of their children in group 1; 13 of the children (9%) were also positive for *H. pylori* stool antigen (HpSA). However, IgG antibodies were present in only 2 of the 10 index parents in group 2. One of their spouses and one of their children had a positive antibody response. All of their children had negative stool antigen test results.

**CONCLUSION:**

*H. pylori* infections exhibit intrafamilial clustering. Parental infection, age ≥ years and having three or more siblings are the major risk factors for *H. pylori* infection in children.

## INTRODUCTION

*Helicobacter pylori (H. pylori)* is the causative agent of peptic ulcer disease and chronic gastritis. It also underlies gastric mucosa-associated lymphoid tissue lymphoma and gastric cancer. The estimated prevalence is almost 70% in developing countries, and 30-40% in the United States and other industrialized countries.^1^ In a recent study conducted in Turkey, the prevalence of *H. pylori* infection was reported to be 82.5% in the adult population.^2^ In developing countries, it is markedly more prevalent at younger ages than it is in developed countries.^3^

Contact with *H. pylori* occurs usually during the first decade of life. *Helicobacter* seropositivity has been found to increase with age.^4^ It has been reported that more than 30% of subjects acquired infection before the teenage period.^5^ Ertem et al. investigated the age-related prevalence of *H. pylori* infection in a group of healthy children. They found that one in five of the children became infected before reaching four years of age and that one in two of the children aged under 11 years was infected.^6^ Transmission is most likely to occur person-to-person, and through fecaloral and oral-oral routes. Low socioeconomic status, poor environmental conditions and living in a crowded house have all been correlated with higher prevalence rates. Better hygiene practices and less household overcrowding have contributed to the decline in prevalence over the last decade.^7^

There are several invasive and noninvasive methods for diagnosing *H. pylori* infection.^8^,^9^ Specific endoscopic findings, the rapid urease test (RUT), histological assessment and gastric tissue cultures all contribute towards making an accurate diagnosis. Molecular methods may be used to prove the presence of *H. pylori*. Noninvasive methods include the urea breath test (UBT), serological tests and stool antigen testing.^10^,^11^ Detection of *H. pylori* antigen in stools provides evidence of active infection. This has been found to be highly concordant with the 13-carbon urea breath test (^13^C-UBT).^12^ The stool antigen test is useful for primary diagnosis as well as for assessment of eradication following therapy.^13^

In this study, we determined the *H. pylori* status of family members, to reveal intrafamilial concurrent infection.

## METHODS

### Study design, setting and ethics

This was a cross-sectional analytical study, conducted in a tertiary care hospital in Istanbul in Turkey. The study was approved by the institution’s Internal Review Board (18/12/2012-174). The procedures followed were in accordance with the ethical standards of the Helsinki Declaration and its revisions. Informed consents were obtained for participation in the study.

### Patient population

All adult consecutive patients with gastroduodenal symptoms who underwent gastroscopy were recruited for this study because this procedure provided a definitive diagnosis of *H. pylori* infection in the index case. These patients were enrolled in the study provided that their spouses and children also agreed to enter the study.

### Investigations for *H. pylori*

The presence of *H. pylori* was determined using RUT (CLOtest) and gastric histological evaluations on biopsy specimens collected by means of endoscopy. Blood samples were drawn from all patients, their spouses and children. Serum samples were stored at -20 °C and were assayed for anti-*Helicobacter* immunoglobulin G (IgG) antibodies (anti-Hp) using a micro enzyme-linked immunosorbent assay (ELISA) (Premier *H. pylori*, Meridian Diagnostics, Inc., Ohio, USA). The assays were performed in accordance with the manufacturer’s instructions. Stool samples were obtained only from the children and were frozen at -20 °C. *H. pylori* antigen was determined from the stool specimens using the Premier Platinum *H. pylori* stool antigen (HpSA) enzyme immunoassay (Meridian Diagnostics, Inc., Ohio, USA) as recommended by the manufacturer. Almost all of them were collected in the hospital. Only a few samples were obtained at home, and these were transferred in cold packs.

### Statistics

The data were analyzed using the Statistical Package for the Social Sciences (SPSS) statistical software (SPSS Inc., Chicago, IL, USA). The chi-square test and Fisher’s exact test were used. P ˂ 0.05 was considered statistically significant.

## RESULTS

Fifty adult patients who underwent gastroscopy (index parents), their spouses (n = 50) and their children (n = 159) were enrolled in the study. Two groups were set up based on the infection status of the index parents: Group 1 consisted of the 40 patients with documented *H. pylori* infection (both RUT and histological evaluations revealing positive results), along with their spouses and children. Group 2 included the remaining 10 patients who underwent endoscopy that did not confirm the presence of *H. pylori* infection, along with their spouses and children. The demographic characteristics of the sample are summarized in **Table 1**.

**Table 1.** Demographic characteristics of the sample

		**Age (years)**		**Gender n (%)**	
	**n**	**Mean ± SD**	**Age range**	**Female**	**Male**
Group 1*					
Parents	80	36.4 ± 0.6	25-51	40 (50)	40 (50)
Children	140	7.7 ± 0.2	1.5-14	73 (52.1)	67 (47.9)
Group 2**					
Parents	20	34.8 ± 0.9	29-44	10 (50)	10 (50)
Children	19	4.4 ± 0.3	2-6	8 (42.1)	11 (57.9)
**Infected parents**	81	36.4 ± 0.6	25-51	41 (50.6)	40 (49.4)
**Non-infected parents**	19	34.8 ± 0.9	29-44	9 (47.4)	10 (52.6)
**Infected children**	131	7.9 ± 0.3	2-14	71 (54.2)	60 (45.8)
**Non-infected children**	28	4.5 ± 0.2	2-6	10 (35.7)	18 (64.3)
**Total study population**					
Parents	100	36 ± 0.5	25-51	50 (50)	50 (50)
Children	159	7.3 ± 0.2	1.5-14	81 (50.9)	78 (49.1)

SD = standard deviation. *Group 1 included the 40 index parents with documented *H. pylori* infection, their spouses and their children; **Group 2 consisted of the 10 index parents who underwent endoscopy revealing negative results for *H. pylori*, their spouses and their children.

The index parents in group 1 were all seropositive. Anti-Hp IgG was found in 38 of their spouses (n = 40) and 130 of their children (n = 140). Stool antigens were detected in only 13 of these children, who were all seropositive. In contrast, anti-Hp was demonstrated in only two of the 10 index parents in group 2; one of their spouses (n = 10) and one of their children (n = 19) also had IgG antibodies. Stool testing for *H. pylori* antigen revealed negative results in all of the children in group 2 (**Table 2**).

**Table 2.** Presence of *H. pylori* (Hp) immunoglobulin G (IgG) antibodies and stool antigen in the family members in relation to the *H. pylori* status of the index parent who underwent endoscopy for gastroduodenal symptoms

	**Group 1**		**Group 2**		**P**
	**n**	**%**	**n**	**%**	
**Anti-Hp, index parent (n = 50)**					
+	40	80	2	4	˂ 0.01
-	0	0	8	16	
**Anti-Hp, spouses (n = 50)**					
+	38	76	1	2	˂ 0.01
-	2	4	9	18	
**Anti-Hp, children (n = 159)**					
+	130	82	1	1	˂ 0.01
-	10	6	18	11	
**HpSA, children (n = 159)**					
+	13	8	0	0	> 0.05
-	127	80	19	12	

HpSA = *H. pylori* stool antigen.

The children were evaluated for the risk factors of *H. pylori* infection. The risk of *H. pylori* infection was higher if both parents were infected and with increasing age among the children and greater numbers of siblings (**Table 3**). Children with positive test results (serological tests and/or positive stool antigen test) were asked about any symptoms of *H. pylori* infection (abdominal pain, dyspeptic signs and gastrointestinal bleeding). All of them were symptom- free.

**Table 3.** Risk factors for *H. pylori* (Hp) infection in children. *H. pylori* infection was determined through a positive test result for either anti-Hp or *H. pylori* stool antigen (HpSA)

	**Children with *H. pylori* infection (n = 131)**	**Children without *H. pylori* infection (n = 28)**	**OR**	**95% CI**
**Infection status of the parents**				
Both parents infected	123	3	128.1	31.8-516.9
Only one or no parents infected	8	25		
**Age of children (years)**				
Age ≥ 7	83	12	2.3	1.0- 5.3
Age ˂ 7	48	16		
**Sex of children**				
Girl	61	14	0.9	0.4-2.0
Boy	70	14		
**Number of siblings**				
≥ 3	106	1	114.5	14.8-883.0
˂ 3	25	27		

OR = odds ratio; CI = confidence interval.

## DISCUSSION

Transmission of *H. pylori* from infected family contacts has become a subject of research through observations of infection in more than one family member.^14^-^15^ These studies have differed regarding their study designs (longitudinal or cross-sectional) and study populations (community-based or focusing on the families of *H. pylori*-positive patients). Most of the studies have been carried out either on families randomly selected from the general population or on the family members of children in certain age groups. On the other hand, some studies have approached this topic from the starting point of *H. pylori*-positive patients or patients with gastroduodenal symptoms and have then investigated their families. The present study was conducted among patients who had been referred to an adult gastroenterology clinic, and we subsequently assessed the infection status of their family members.

Various diagnostic tests have been used in research exploring the intrafamilial transmission of *H. pylori*. These tests have comprised anti-Hp, UBT, HpSA and RUT, along with molecular methods such as the polymerase chain reaction (PCR).^16^-^24^ In our study, the infection status of the index parents was definitively determined by means of RUT and histological evaluations. The spouses and children were tested via serological tests, while a stool antigen kit was also provided for the children.

The most important finding from this study was the high seropositivity observed among the children (82.4%). The previously reported local seropositivity rates were 33%, 49.5% and 43.9% respectively.^5^,^6^,^14^ The considerably high level of seropositivity found here can be attributed to the fact that 93.9% of those children had both parents infected with *H. pylori* who had been suffering gastroduodenal symptoms. This increases the chance of transmission. Lower rates might have been observed in community-based studies.

The key role of parents in transmitting *H. pylori* to their children has been shown in various population-based studies.^14^-^21^ Dominici et al. assessed the infection rate of children in relation to their parents’s infection status, using IgG antibodies. They reported that children whose parents were both seropositive had double the risk of being infected, compared with those whose parents were both seronegative.^15^ In another study, the infection status was determined using UBT among children and salivary IgG antibodies among their parents. It was concluded that infected parents, especially mothers, played a key role in the transmission of *H. pylori* to their children.^16^

Weyermann et al. conducted a prospective birth cohort study to investigate the acquisition of *H. pylori* infection in early childhood and to clarify the role of parental infection status in the transmission of *H. pylori* to children. Mothers who gave birth to a healthy child, their partners and their other children were included in the study. The presence of active infection of the mothers with *H. pylori* was determined using UBT. The infection statuses of the fathers at the beginning and of their children at the ages of one, two and three years were determined by means of HpSA. These authors claimed that an infected mother was likely to be the main source for *H. pylori* infection among their children. Kissing, shared use of spoons, cleaning pacifiers (dummies) or teats of feeding bottles in the mouth and sharing a bed may facilitate its spread.^17^

Evidence of mother-child transmission of infection was also reported in an article by Escobar and Kawakami.^18^ Nahar et al. screened 55 families for *H. pylori* using a stool antigen test. Those who tested positive were further evaluated through culturing biopsy material or gastric juice. These authors then performed PCR-based random amplified polymorphic deoxyribonucleic acid (DNA) (RAPD) fingerprinting to explore the genetic diversity of *H. pylori* within families. They observed shared genotypes in the paired strains from mothers and children and concluded that vertical transmission was the most probable route of transmission.^19^

Early diagnosis and prompt treatment of patients might prevent the spread of infection to spouses and children. In the present study, we observed that parental infection was a risk factor for children. However, the mothers’ role in the transmission could not be documented, since the proportions of infected mothers and fathers among the infected parents were not significantly different and both spouses were infected in most cases.

Several studies on intrafamilial transmission have been conducted among the parents and siblings of children who either had been referred due to gastroduodenal symptoms or had previously been found to present *H. pylori*-positive status. Drum et al. demonstrated that parents of *H. pylori*-positive children were more likely to have a positive serological response than were the comparably-aged parents of noncolonized children, thus indicating the existence of intrafamilial clustering of *H. pylori* infection.^22^

In another study, *H. pylori* infection was investigated by means of upper gastrointestinal endoscopy and UBT in 100 children with upper gastrointestinal symptoms. UBT was performed on all family members of each index patient. The prevalence of *H. pylori* infection was significantly higher among the families of infected children.^23^ After analyzing 35 children with *H. pylori* gastritis and their family members, Yokota et al. concluded that intrafamilial infection was the dominant transmission route.^24^

All of the above studies focused on index pediatric patients. Our study differed from these through focusing on parents with gastroduodenal symptoms and concentrating on the infection status of their family members. We demonstrated that among the 126 children whose parents were both infected, 123 had a positive test result for either anti-Hp or HpSA. On the other hand, when the parents were not infected, their children were not infected either (**Table 3**). This reflects exact clustering of the infection in the family, thus suggesting that close personal contact facilitated transmission. These families belonged to a middle-class socioeconomic group, and there seemed to be no other risk factor attributable to their living conditions. All of their homes had a water supply from the city and were connected to the sewer system.

There have been reports mentioning higher prevalence of infection among spouses.^25^-^28^ Brenner et al. assessed the clustering of *H. pylori* infection among healthy couples. Active infection was measured by means of the urea breath test and HpSA. Their results supported the hypothesis that spouse-to-spouse transmission had a major role in *H. pylori* infection.^29^ However, several years after that report, the same researchers assessed clustering of *H. pylori* infections in both high and low-prevalence population subgroups. They stated that spouse-to-spouse transmission of infection was unlikely to be of relevance in low-prevalence population groups, although clustering of infection was observed in high-prevalence population groups.^30^

Horizontal transmission of *H. pylori* may play an important role in developing countries. However, improved sanitation and quality of life have markedly reduced the risk in developed countries, which has thus enhanced the role of intimate contact in intrafamilial infection. In the present study, we observed seropositivity in 38 spouses of the 40 *H. pylori*-positive index patients (95%). On the other hand, only one out of the 10 *H. pylori*-negative index patients had a seropositive spouse.

Children play a role in spreading *H. pylori*. Infection may be transmitted among siblings. We documented that sibship size was a risk factor (**Table 3**). 99% of the children were infected when the number of siblings was ≥ 3, whereas 48% were infected when the number of siblings was ˂ 3. The role of siblings in the transmission of infection has previously been reported.^23^,^31^-^33^ Infection among siblings may be of more relevance in high-prevalence countries. Transmission probably occurs in early childhood, from older to younger siblings.^31^,^32^

The prevalence of infection among the children was quite high, with an overall seropositivity rate of 82%. However, only 13 children who were seropositive were also positive for HpSA. This ratio (8%) was unexpectedly low. The HpSA test has been found to be a reliable method for diagnosing and following up *H. pylori* infection. It is considered useful for screening and monitoring.^34^ In order to perform diagnostic tests other than serological tests, waiting two weeks after the end of proton pomp inhibitor therapy and four weeks after the end of antibiotic therapy is recommended.^3^,^34^ Prior antibiotic therapy or spontaneous elimination of *H. pylori* infection^35^ may explain the high seropositivity rate (82% of the children in the present study) alongside the low positivity rate for stool antigen (8%) in those children. Constipation, presumably due to degradation of *H. pylori* antigens, may also contribute to this result.

*H. pylori*-positive patients pose a risk of transmission of infection to their other family members. We observed that if one of the parents was infected with *H. pylori*, the risk of infection was significantly higher for their spouses and children. When both parents were infected, their children had a higher rate of infection than did children with only one or no parents infected. Crowded living conditions (number of siblings > 3) increased the risk. These findings support the notion that transmission occurs from person to person, through close contact, and that it occurs during childhood.

One limitation of the present study is that we did not prove that a single *H. pylori* strain caused the intrafamilial clustering in each family. Nonetheless, presence of a single strain would not constitute sufficient evidence of person-to-person transmission. The shared strain indicates only a common source, which may be an environmental factor as well. However, our patients had access to a water supply from the city and adequate sanitation facilities.

The joint European Society for Paediatric Gastroenterology Hepatology and Nutrition (ESPGHAN)/North American Society for Pediatric Gastroenterology, Hepatology and Nutrition (NASPGHAN) guidelines do not recommend starting treatment based on screening for the presence of *H. pylori* infection by means of a noninvasive test applied to children (recommendation 2c). Instead, they recommend testing for *H. pylori* among children with gastric or duodenal peptic ulcer disease (recommendation 3).^34^

Presence of *H. pylori* is a risk factor for duodenal ulcers in children.^36^ Considering the fact that it is highly transmitted within family settings, presence of parental *H. pylori* infection should prompt physicians to ask children about any gastroduodenal symptoms compatible with peptic ulcer disease. Children with positive symptoms should be further evaluated for peptic ulcer disease.

There is a need to determine which symptoms should be interpreted as indicative of the presence of peptic ulcer disease. Hernandez et al. performed upper gastrointestinal endoscopy due to suspicion of peptic disease when at least one of the following manifestations was observed: hematemesis; chronic epigastric pain or nocturnal awakening with abdominal pain; chronic vomiting associated with eating; suspected peptic ulcer disease relapse; and recurrent abdominal pain in children with a first-degree relative with peptic ulcer disease. *H. pylori* infection was confirmed in 56.1% of the children and duodenal ulcer was diagnosed in 32 patients (13.5%).^37^ Guariso et al. declared that upper gastrointestinal endoscopy was appropriate for cases with a family history of peptic ulcer and/or *H. pylori* infection, in individuals older than 10 years of age, with dyspeptic symptoms persisting for more than six months that were severe enough to affect activities of daily living.^38^ Thus, analysis on the cost-effectiveness of further investigation of the patients with gastroduodenal symptoms compatible with peptic ulcer disease and a family history of *H. pylori* infection could form the subject of a further study.

## CONCLUSION

We observed concurrent intrafamilial *H. pylori* infection. It may have been transmitted within the family. Parental infection, age ≥ years and having three or more siblings are the major risk factors for *H. pylori* infection in children. Infection of parents puts children at risk. The risk increases with age and number of siblings.
